# Evaluation of the Effects of the Tritordeum-Based Diet Compared to the Low-FODMAPs Diet on the Fecal Metabolome of IBS-D Patients: A Preliminary Investigation

**DOI:** 10.3390/nu14214628

**Published:** 2022-11-02

**Authors:** Giusy Rita Caponio, Giuseppe Celano, Francesco Maria Calabrese, Giuseppe Riezzo, Antonella Orlando, Benedetta D’Attoma, Antonia Ignazzi, Mirco Vacca, Annalisa Porrelli, Valeria Tutino, Maria De Angelis, Gianluigi Giannelli, Francesco Russo

**Affiliations:** 1 National Institute of Gastroenterology “S. de Bellis” Research Hospital, 70013 Castellana Grotte, Italy; 2 Department of Soil, Plant and Food Science, University of Bari Aldo Moro, 70126 Bari, Italy

**Keywords:** diet, functional gastrointestinal disorders, irritable bowel syndrome, metabolome, short-chain fatty acids, Tritordeum, volatile organic compounds

## Abstract

Since irritable bowel syndrome (IBS)—a common gastrointestinal (GI) disorder—still lacks effective therapy, a nutritional approach may represent a practical alternative. Different reports demonstrated that a low-fermentable oligosaccharides, disaccharides, monosaccharides and polyols (FODMAPs) diet (LFD) reduces symptoms in IBS with diarrhea (IBS-D) patients, also inducing beneficial pathophysiological and biochemical modifications. More recently, diets with alternative cereals having a different gluten composition, such as tritordeum, have also been considered (TBD). We investigated the impact of TBD and LFD on the fecal metabolome composition in 38 IBS-D patients randomly allocated to the two diets for 12 weeks. Summarily, at baseline, the profile of fecal volatile organic compounds (VOCs) of IBS-D patients was not significantly different in the two groups. After treatment, significant changes were observed in the two groups regarding the VOCs content since some of them increased in the TBD group (namely, decanoic acid), whereas others (i.e., nonanal and ethanol) increased in the LFD one. Further, at baseline, short-chain fatty acids were positively related to inflammation and showed a significant decreasing trend after both diets compared to baseline values (namely, acetic and propanoic acid). Preliminary results from this pilot study suggest a potential positive intervention of TBD and LFD affecting the fecal metabolome composition in IBS-D patients.

## 1. Introduction

Irritable bowel syndrome (IBS) is a common functional gastrointestinal disorder (FGID) with a prevalence of 15% to 20% [1]. Based on their intestinal habits, IBS patients may be categorized into four different groups, namely IBS with diarrhea (IBS-D), IBS with constipation (IBS-C), IBS with alternating diarrhea and constipation (IBS-M), and undefined IBS (IBS-U) [2].

Due to the lack of reliable information on the etiology, therapies range from supplements, laxatives, or prokinetics for patients primarily suffering from constipation to the use of drugs [3]. However, the initial treatment for IBS is lifestyle education and an adequate diet since many patients relate their symptom profiles to ingesting specific foods. In the last decade, research has focused on the association between IBS and consuming foods rich in fermentable oligosaccharides, disaccharides, monosaccharides, and polyols (FODMAPs) [4]. Limiting the intake of these indigestible short-chain carbohydrates can significantly improve IBS symptoms [5]. This evidence is supported by recent reports on the effects of a low-FODMAPs diet (LFD) in patients with IBS-D [6,7]. Symptom reduction was associated with nutritional, anthropometric, and biochemical variables. In addition, changes in intestinal permeability and some modulators of intestinal barrier function and integrity (e.g., zonulin and diamine oxidase (DAO)) accompanied the reduction of symptoms. However, LFD compliance is problematic and requires ongoing nutritional support. As a result, new, helpful dietary approaches, including innovative cereals, have more recently gained interest. Notably, tritordeum, a grain derived from a cross between durum wheat and wild barley [8,9], has shown beneficial, healthy properties, suggesting its potential use in the diet to treat non-celiac wheat sensitivity [10].

Tritordeum differs in its gluten components by a specific gliadin content and a significantly reduced concentration of immunogenic gluten peptides. This grain is also rich in fiber and natural antioxidants such as lutein, an antioxidant carotenoid that protects the eyes and skin, and phenolic compounds that have been shown to protect against colon cancer [11]. Notably, tritordeum contains more protein and fewer carbohydrates than other grains. In addition, it contains high amounts of fructans, compounds with prebiotic properties that help maintain healthy homeostasis of the gut microflora [12].

As recently described by our group [13], patients with IBS-D who received a tritordeum-based diet (TBD) for 12 weeks experienced significant reductions in gastrointestinal (GI) symptoms. This effect can be attributed to the overall improvement of the GI barrier, evidenced by decreased intestinal permeability (IP) and favorable changes in intestinal barrier integrity, along with reduced mucosal inflammation and fermentative dysbiosis. Furthermore, a previous study highlighted that seven days of tritordeum bread did not significantly alter the gut microbiota composition in non-celiac wheat-sensitive patients. Only a few changes were observed in some butyrate-producing bacteria [14].

Despite many metagenomic data, the functional dynamics of gut microbiota in FGIDs, particularly IBS-D, remain largely unexplored. 

The microbiota produces a variety of metabolites from dietary products with consequences for host health and pathophysiology, significantly affecting gut barrier function and immune responses. 

Since microbial homeostasis and gut barrier health are closely related, attempting to maintain a probiotic state in the gut microbial ecosystem is essential to prevent gut dysbiosis and microbial imbalance [15,16]. In line with this concept, our study aimed to evaluate the effect of 12 weeks of TBD and LFD administration on the fecal metabolomic profile of patients with IBS-D.

## 2. Materials and Methods

### 2.1. Subject Recruitment and Study Design

This pilot study was conducted at the National Institute of Gastroenterology “S. de Bellis” Research Hospital between January 2018 and September 2020. Patients referred to the Functional Gastrointestinal Disorders Research Unit between 18 and 65 years and diagnosed with IBS-D according to the Rome IV criteria were recruited. Exclusion criteria were: organic diseases with a diagnosis of structural abnormality of the GI tract; metabolic disturbances; mental illness; and having already followed a diet (i.e., vegan diet, gluten-free diet, low-carbohydrate high-fat diet, or LFD). Other exclusion criteria were based on the occurrence of antibiotic therapy, probiotic agents, other medications causing abdominal pain, selective serotonin reuptake inhibitors and other antidepressant drugs, and drugs for treating IBS in the last two weeks before evaluation. In the case of lactose intolerance, the lactose-reduced diet was allowed as long as patients agreed to keep this intake constant during the study period. Another exclusion criterion was patients’ unwillingness to change their current diet.

At baseline, all subjects underwent specific analyses to exclude celiac disease (celiac-specific serum IgA and IgG titers), wheat allergy (total and wheat-specific immuno-globulin E antibodies), and cutaneous wheat sensitization (prick test).

All subjects received the assigned diet treatment for 12 weeks. Before the diet started, at baseline (T_0_), patients underwent a physical examination by a gastroenterologist and an interview with a nutritionist. The IBS Severity Scoring System (IBS-SSS) symptom questionnaire (see “Symptom profile” section) and fecal samples for analyzing fecal metabolome were collected at T_0_ and after 12 weeks of the assigned diet (T_1_).

**Figure 1:**
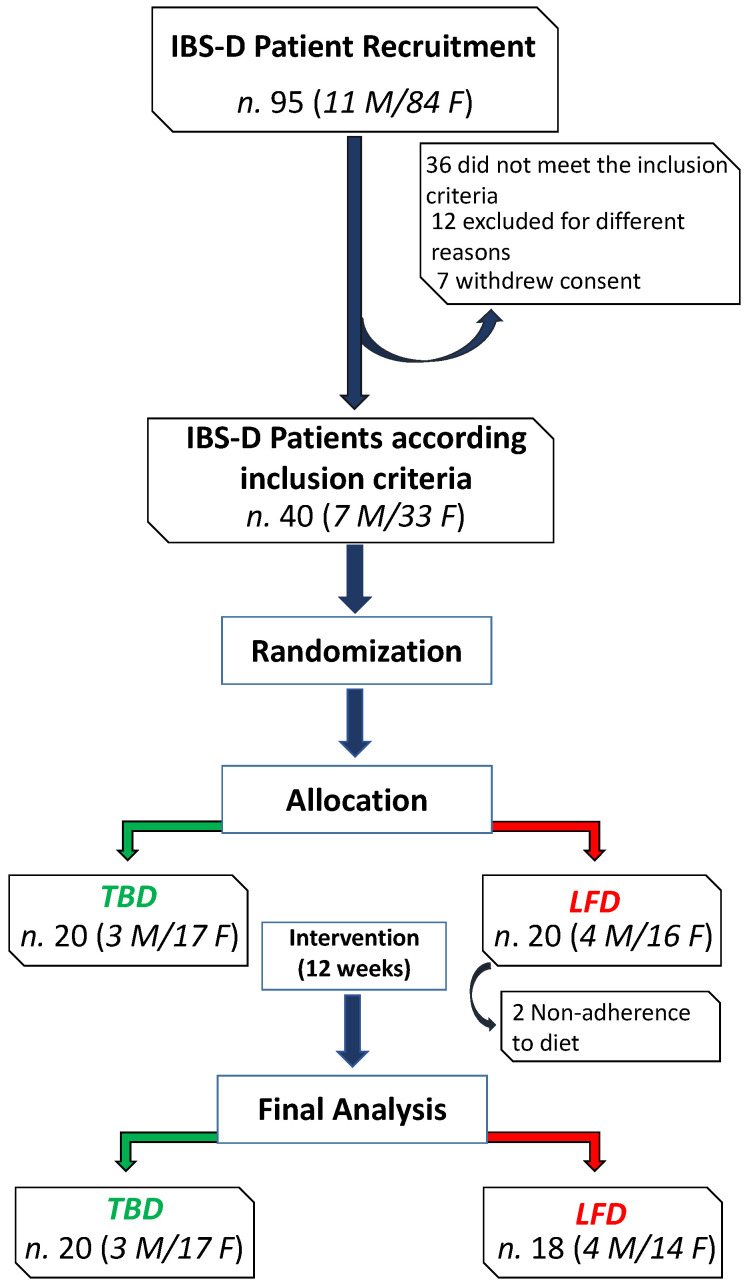
The CONSORT flowchart of the study. Abbreviations: IBS-D, irritable bowel syndrome with diarrhea; LFD, low-FODMAP diet; TBD, tritordeum-based diet.

Figure 1 depicts the flowchart of the study. The initial cohort comprised 95 IBS-D patients, 11 males (M) and 84 females (F), aged 48 ± 11 years. Thirty-six patients did not meet the inclusion criteria, twelve were excluded for different reasons (e.g., declared difficulty in following the proposed diet, work or study reasons, transfer to another city), and seven withdrew their consent. Thus, forty patients (7 M/33 F) reached the diet-assignment phase: 20 patients (3 M/17 F) were allocated to the TBD group and 20 patients (4 M/16 F) to the LFD one, but two female patients of the latter group dropped out for non-adherence to diet. Overall, 38 IBS-D patients completed the study and the metabolomic analysis.

The present study was part of broader research aimed at comparing alternative nutritional strategies based on a low-FODMAPs diet and usual dietary advice administered to IBS-D patients. The study was approved by the Scientific Committee of IRCCS “S. de Bellis” and the Ethics Committee of IRCCS “Ospedale Oncologico–Istituto Tumori Giovanni Paolo II”, Bari, Italy (N. 274/C.E. 12.12.17). The trial has been registered on http://www.clinicaltrials.gov–NCT03423069 (accessed on 22 March 2022). All patients gave full written informed consent.

### 2.2. Characteristics of Tritordeum-Based Diet and Low-FODMAPs Diet

Throughout the study, both groups followed a controlled diet under the supervision of trained nutritionists. At baseline, patients completed a food diary to evaluate their usual diet and calculate energy requirements and received general recommendations about performing physical activity.

Nutritionists examined the completed food diaries to calculate the daily energy intake of carbohydrates, lipids, proteins, and dietary fibers. A special software (Progetto Dieta v. 2.0, www.progettodieta.it accessed 18 January 2022) was used [6,7,13]. 

The diets were prepared by matching basal metabolism and daily energy consumption with anthropometric data for all patients to assign suitable and tailored dietary regimens. The daily intake of macronutrients (50% carbohydrates, 30% lipids, and 20% proteins) was calculated by dedicated software (Nutrigeo Software 8.6.0.0, Progeo Medical, Centobuchi di Monteprandone, Ascoli Piceno, Italy).

During the study, patients kept food diaries to assess their energy intake and expenditure. The diary contained the quantities (in grams) and descriptions of foods eaten each day for three meals (breakfast, lunch, and dinner) and two snacks (morning and afternoon). As concerns the two intervention diets, each patient belonging to the TBD group consumed all the food items (bread, pasta, “taralli” (local salty biscuits), and breakfast biscuits) prepared using tritordeum flour exclusively, as reported elsewhere [7]. Tritordeum products were freely provided to each patient throughout the study with a monthly schedule. Patients in the LFD group consumed this controlled diet limiting the intake of foods containing fermentable oligosaccharides, monosaccharides, disaccharides, and polyols [6]. To ensure proper adherence to LFD, a booklet detailing which foods were allowed and which were avoided or reduced was provided to each participant.

At the end of the study, patients also had to deliver a compiled IBS Diet Adherence Reporting Scale (IDARS) consisting of five questions to assess their compliance with the recommended diet, with scores ranging from 1 to 5 for each item. Good adherence to the diet was confirmed by a total score of at least 20 [7].

### 2.3. Symptom Profile

The symptom profile in IBS-D patients was evaluated using the IBS-SSS total score [17] to have a single parameter of symptom severity. The IBS-SSS total score is a composite score of five items (the severity of abdominal pain, the frequency of abdominal pain, the severity of abdominal distension, dissatisfaction with bowel habits, and the impact of symptoms on quality of life). It describes the severity of IBS symptoms based on the 100-point visual analog scale (VAS) to provide a total score between 0 and 500. Scores indicated mild, moderate, and severe cases, ranging from 75 to 175, 175 to 300, and >300, respectively. Healthy subjects have a score below 75, and patients having scores in this range could be considered in remission and thus were excluded from the study.

### 2.4. Fecal Metabolome

Fecal samples were collected from patients to assess volatile organic compound (VOC) profiles. The best extraction efficiency was obtained by performing the procedure described by Lauriero et al. [18] with slight modifications. Briefly, a 1 g stool sample added with 10 µL of 4-methyl-2-pentanol (9.9 µg/g final concentration) as internal standard (IS) was placed in a 10 mL glass vial and sealed with a polytetrafluoroethylene (PTFE)-coated silicone rubber septum, then incubated for 10 min, and equilibrated at 40 °C. The extraction of VOCs, the fiber information, and the autosampler used agreed with those indicated in the paper by Vitellio et al. [19].

VOCs were thermally desorbed by immediately transferring the fibers to the heated inlet (220 °C) of a Clarus 680 (Perkin Elmer, Beaconsfield, UK) gas chromatograph equipped with an Rtx-WAX column (30 m × 0.25 mm id, 0.25 µm film thickness) (Restek Corporation, Bellefonte, PA, USA) and coupled to a Clarus SQ8MS (Perkin Elmer, Beaconsfield, UK) with source and transfer line temperatures maintained at 250 and 210 °C, respectively. Peak identification analysis was performed on each chromatogram using the National Institute of Standards and Technology 2008 (NIST) library (Gaithersburg, MD, USA). VOC identification used a peak area threshold of >1,000,000 and a match probability of 85% or higher, followed by manual visual inspection of fragmentation patterns when required. The concentration of VOCs was expressed in µg/g of IS.

### 2.5. Quantitative Analysis of Targeted VOCs in Fecal Samples

A targeted analysis of short-chain fatty acids (SCFAs) and branched-chain fatty acids (BCFAs) was conducted to evaluate the differences among groups. The standard curve was made with a pure standard of acetic acid, butyric acid, propionic acid, isobutyric acid, and isovaleric acid (Sigma-Aldrich, St. Louis, MO, USA) and IS (final concentration of 1 mg/L). Total ion current mode was used to obtain typical ions with a special mass-to-charge ratio of each compound, and then, selective ion monitoring (SIM) mode was used to evaluate the concentration of each compound [20]. The relative peak of SCFAs in the fecal sample was integrated, and the absolute concentration (ppm) of SCFAs in the fecal sample was calculated by the calibration curve equation.

### 2.6. Statistics

A per-protocol analysis was performed using only participants who completed all testing points. Non-parametric tests were performed to avoid the assumption of normal distribution because of the small number of patients studied. Specifically, the Mann–Whitney test was performed to compare the between-group differences, and Wilcoxon rank-sum test was used to compare the within-group differences (pre- and post-treatment data). Spearman correlation analysis was performed to determine the putative correlations between symptoms, which was expressed as IBS-SSS total score, and untargeted VOC concentrations. A *p*-value less than 0.05 was considered statistically significant. Unless otherwise specified, all results were expressed as mean ± standard deviation (SD). The statistic packages were Sigma Stat 11.0 (Systat Software, Inc., San Jose, CA, USA) and GraphPad Prism 5 (GraphPad Software Inc., La Jolla, CA, USA).

## 3. Results

### 3.1. Cohort, Baseline Characteristics and Diet

As shown in Table 1, at baseline, no significant differences were detected between IBS-D patients receiving TBD and LFD treatment according to age, sex, body mass index (BMI), and abdominal and waist circumference.

The patients’ daily nutritional information recorded at the start and the end of the two diets is shown in Table 2. As reported below, significant differences (*p* < 0.05, data analyzed by Wilcoxon signed-rank test) were found in both diets between baseline values (T_0_) and those recorded at the end of the study (T_1_). On the contrary, no significant differences between the two diets were found when compared at the same time points (*p* > 0.05, data analyzed by the Mann–Whitney test).

### 3.2. Symptom Evaluation

**Figure 2:**
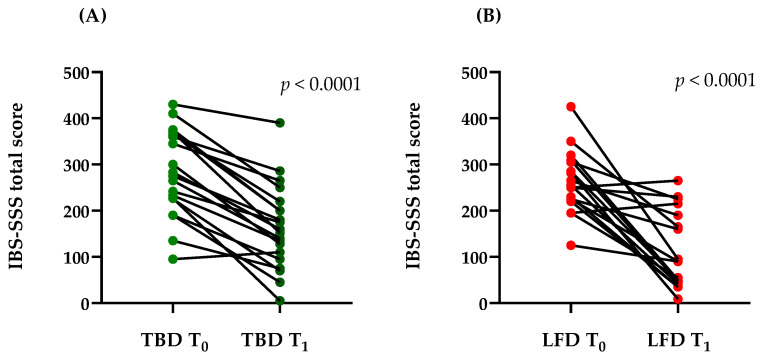
Total IBS-SSS score before (T_0_) and after (T_1_) TBD (**A**) and LFD (**B**), showing a clear reduction of the mean IBS-SSS total score after diet from moderate-to-mild range (284.25 ± 92.82 vs. 164.05 ± 90.73 and 261.72 ± 66.99 vs. 113.78 ± 82.48; TBD and LFD, respectively). Data are expressed as mean ± standard deviation (SD). Statistical analysis: Wilcoxon rank-sum test. Abbreviations: LFD, low-FODMAPs diet; TBD, tritordeum-based diet; IBS-SSS, irritable bowel syndrome (IBS) Severity Scoring System.

Figure 2 reports the total scores of the IBS-SSS questionnaire in the LFD and TBD groups. Mean scores at baseline indicated that all the IBS-D patients, irrespective of diet allocation, belonged to moderate IBS-SSS group (284.25 ± 92.82 vs. 261.72 ± 66.99; TBD and LFD, respectively) without any significant differences between the two groups (*p* = 0.3806).

IBS symptoms were markedly reduced after 12 weeks of both diets. In detail, the total score decreased by 42.78% after TBD (*p* < 0.0001) and by 56.51% after LFD (*p* < 0.0001). After the diets, the IBS-SSS total score was 164.05 ± 90.73 and 113.78 ± 82.48 (TBD and LFD, respectively), showing a slight difference in favor of the LFD but without a statistical significance (*p* = 0.1004).

### 3.3. Fecal Metabolome Analysis

**Figure 3:**
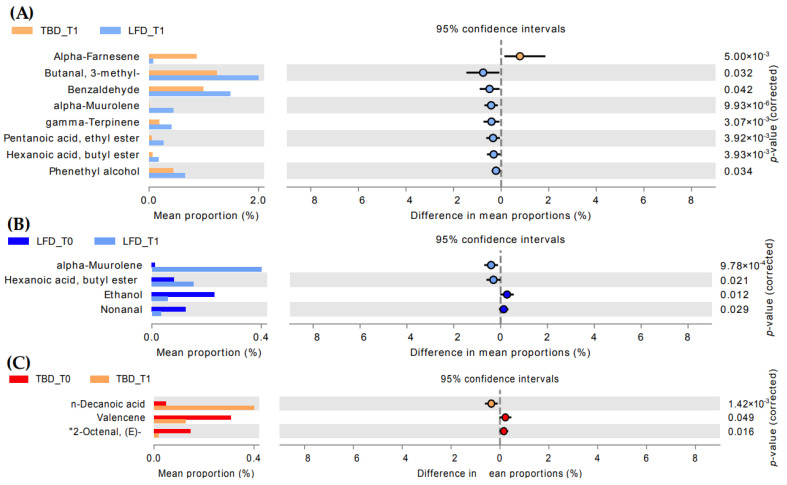
Extended error bar-plot of statistically significant volatile organic compound differences (Wilcoxon rank-sum’s test) resulted from pairwise comparison between TBD and LFD groups after treatment (**A**) and within each group: (**B**) = LFD and (**C**) = TBD. Abbreviations: LFD, low-FODMAPs diet; TBD, tritordeum-based diet.

An objective comparison of VOC profile in fecal samples from IBS-SSS patients at the baseline and after 12 weeks of diet regimen was performed based on qualitative and quantitative differences in VOCs using headspace solid-phase microextraction coupled to gas chromatography–mass spectrometry (HS-SPME GC-MS) analyses. One hundred and twenty-six VOCs were grouped into the following chemical classes: alcohols (7), esters (30), aldehydes (9), phenols (4), ketones (13), organic acids (10), terpenes (19), hydrocarbons (10), indoles (2), furan compounds (2), and lactones (2), and eighteen compounds not belonging to the listed classes were identified (Supplementary Materials Table S1). At baseline, pairwise comparison of diet regimen groups did not show any statistically significant differences, whereas after 12 weeks of treatment, the LFD group was characterized by a higher amount (*p* < 0.05) of aldehydes (3-methyl-butanal and benzaldehyde), terpenes (alpha-muurolene and gamma-terpinene), esters (pentanoic acid, ethyl ester, and hexanoic acid butyl ester), and phenethyl alcohol (Figure 3A). Conversely, TBD was characterized by a higher alpha-farnese amount than LFD (*p* < 0.05).

The differences between time points (T_0_ and T_1_) were also investigated. After treatments, significant differences (*p* < 0.05) were observed for 2-octenal, n-decanoic acid, and valencene in TBD T_1_ compared to the baseline (Figure 3C). Some statistically significant differences were also highlighted in the LFD group (Figure 3B). In detail, an increase of alpha-muurolene and butyl ester of hexanoic acid and a decrease of ethanol and nonanal were observed (*p* < 0.05).

### 3.4. Correlations between IBS-SSS Score and Untargeted VOCs

**Figure 4:**
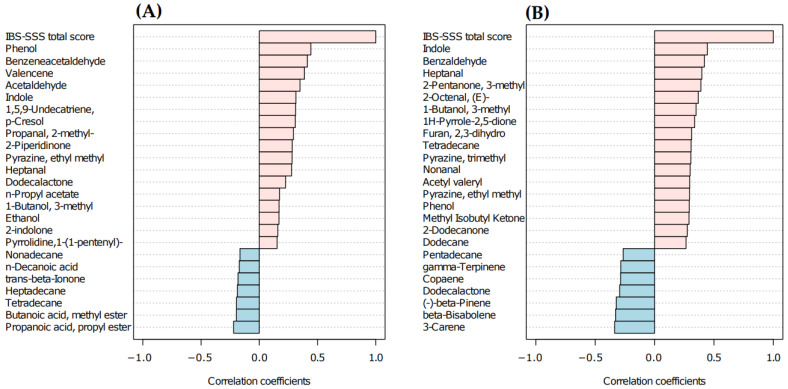
Spearman R-correlation coefficient of top 25 VOCs with IBS-SSS total score in patients administered with TBD (**A**) and LFD (**B**). Positive correlation values are shown in pink, while negative ones are in light blue. Abbreviations: LFD, low-FODMAPs diet; TBD, tritordeum-based diet; VOC, volatile organic compound.

The abundance of untargeted VOCs was correlated with IBS-SSS total score. The investigation was carried out considering sample times (T_0_ and T_1_) in TBD and LFD groups (Figure 4A,B). The Spearman’s rank correlation showed that phenol, indole, heptanal, 3-methyl-1-butanol, tetradecane, and ethyl-methyl-pyrazine were positively correlated with IBS-SSS total score in both groups. Significant negative correlations were assessed for 3-carene, beta-bisabolene, and beta-pinene in the LFD group. Concerning the TBD group, statistically significant positive correlations were observed for phenol, benzeneacetaldehyde, valencene, acetaldehyde, indole, and 1,5,9-undecatriene 2,6,10-trimethyl.

### 3.5. Quantitative Analysis of Targeted VOCs in Fecal Samples

**Figure 5:**
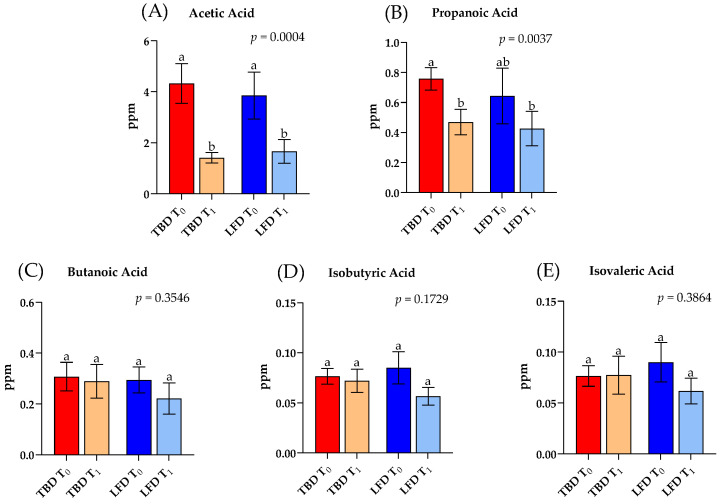
Significant (adjusted *p* < 0.05) concentrations (ppm) of short-chain fatty acids (SCFAs) and branched-chain fatty acids (BCFAs) in headspace fecal samples of IBS-D patients before (T_0_) and after (T_1_) TBD and LFD. Data are expressed as mean (M) ± standard error of the mean (SEM). Statistical differences were obtained by the Kruskal–Wallis test and corrected by Dunn’s multiple Test. Different letters differ significantly (*p* < 0.05). Abbreviations: IBS-D, irritable bowel syndrome with diarrhea; LFD, low-FODMAPs diet; TBD, tritordeum-based diet.

Differences in the fecal levels of SCFAs of IBS-D patients were investigated before and after TBD and LFD for 12 weeks (Figure 5). Targeted analysis of fecal samples highlighted the content in ppm of the main SCFAs (i.e., acetic acid, propanoic acid, butanoic acid, isobutyric acid, and isovaleric acid). Overall, the total amount of SCFAs showed a decreasing trend after both diets compared to baseline values.

Regarding acetic acid (Figure 5A), significant differences were present among groups (*p* = 0.009). According to Dunn’s post hoc test, the administration of TBD and LFD for 12 weeks caused a marked and significant reduction (−67% and −57%; *p* = 0.009 and *p* = 0.030, respectively) in comparison to baseline values.

The analysis of propanoic acid also showed significant differences among groups (*p* = 0.004). In this case, a marked and significant decrease (−38% *p* = 0.031; Dunn’s post hoc test) occurred after treatment with tritordeum (TBD T_1_) compared to TBD T_0_. On the contrary, 12 weeks of LFD decreased by 33% the concentration of propanoic acid compared to LFD T_0_ but without reaching significant differences (*p* > 0.9999; Dunn’s post hoc test) (Figure 5B).

Butanoic acid, isobutyric acid, and isovaleric acid did not show statistically significant differences (*p* > 0.05) among groups (Figure 5C–E).

## 4. Discussion

It is now well-established that diet can affect the intestinal transit time, interact with the gut microbiota, and consequently produce fecal gas, thus directly affecting the development of various symptoms of IBS [21,22]. Further, it has been reported that diet produces different fecal VOCs depending on its type, as different diets have a different impact on intestinal transit and involve changes in the physiology of the colon and the composition of the intestinal microbiota [23,24]. Since GI diseases are associated with a relatively low abundance of microbial flora, the number of secondary metabolites is likely to be reduced. In fact, due to lessened bacterial biodiversity and reduced intestinal transit time, fewer compounds are biosynthesized [25].

Recent observations by our group indicated that both TBD and LFD treatments for 12 weeks significantly ameliorated the symptom profile of IBS-D patients [9,10,16]. These results were once more confirmed in this pilot study, as highlighted by the significant and marked reduction of the IBS-SSS total score after the treatments. As a further step in the attempt of comprehending the basis of the positive effects due to these diets, we aimed to investigate the possible differences in the fecal metabolome of these patients before and after TBD and LFD.

In this study, we performed HD-SPME GC-MS analyses to determine fecal VOC profiles. As expected, our results highlighted that the two groups of intervention (TBD and LFD) did not show significant differences in the metabolomic profile at baseline. On the other hand, we identified VOCs including terpenes (alpha-farnesene, alpha-muurolene, gamma-terpinene), aldehydes (benzaldehyde; butanal, 3-methyl), one alcohol (phenethyl alcohol), and esters (pentanoic acid, ethyl ester, hexanoic acid, butyl ester) that defined the experimental groups divided by diet type (LFD or TDB) at T_1_. Derivative metabolites from BCFAs and aromatic amino acids including aldehydes and phenethyl alcohol were significantly higher in the LFD diet, which was characterized by an increased intake of protein, as shown in our previous work [6]. Although not statistically significant, a slightly higher protein intake was evaluated in LFD. This could explain the significative differences evaluated in this group after treatment compared to TBD. In addition, potential diagnostic biomarkers identified within the VOCs could be represented by benzaldehyde. This compound is produced during inflammation due to oxidative stress and lipid peroxidation caused by the degenerative process affecting the polyunsaturated fatty acids in the cell membrane [26]. Of note, the untargeted VOCs profile showed a lower value of benzaldehyde in TBD T_1_ compared to LFD T_1_. This result was supported by Spearman’s rank correlation that underlined the positive correlation between IBS-SSS total score and benzaldehyde in LFD group.

The fecal metabolome of IBS-D patients undergoing TBD and LFD treatments for 12 weeks showed an increase of the terpene’s metabolites compared to baseline (TBD T_0_ and LDF T_0_, respectively). Specifically, alpha-muurolene—belonging to the class of terpenes—in LFD T_1_ was significant higher compared to the baseline. Previous evidence supported the idea that terpenes are strictly correlated to potentially beneficial implications in conditions of low inflammation, such as IBS-D [27,28]. In fact, it was demonstrated how the abundance of some intestinal microorganisms causing gastrointestinal infections (*Campylobacter jejuni* and *Clostridium difficile*), compared to others, reduces the number of some secondary metabolites (such as terpene metabolites) [29]. Secondary metabolites derived from the microbial metabolism of diet compounds act as prebiotic-like molecules that, in turn, can modulate the growth of specific bacterial strains. Dietary macro- and micronutrients seem to be the main drivers of the metabolic pathways of the intestinal microbiota, affecting, in turn, the fecal metabolomes. Thus, the secondary metabolites showed an anti-inflammatory effect delaying the onset and/or progression of different gastrointestinal pathologies, including ulcerative colitis [30].

A significant decrease of nonanal after treatment was observed in the LFD group. This lipid aldehyde belongs to n-alkenals and is formed in vivo via host or bacterial metabolism or can be derived from the autoxidation of unsaturated fatty acid. Studies supported the proinflammatory effect of this lipid aldehyde classified as n-alkenals through tumor necrosis factor α (TNF-α) up-regulation [31]. In our results, nonanal was positive correlated with IBS-SSS total score in LFD group, suggesting its contribution in low-level induction of chronic inflammation via pro-inflammation cytokines [32]. The evaluated decrease of this compound after LFD could explain the improvement in IBS-SSS total score through the inflammatory response modulation [33]. Moreover, the decrease of ethanol could be positively related to a lower inflammatory response [34].

In the TBD group, some statistically significant differences were found by comparing T_0_ and T_1_. Among these, a significant increase of decanoic acid after treatment was observed. The origin of these medium-chain fatty acids (MCFAs) is predominantly extrinsic, deriving from diet [35]. An ex vivo study on intestinal cells showed how decanoic acid alleviated inflammatory cytokine production (TNF-α and interleukin-6 (IL-6)) and related gene expression (nuclear factor kappa B (NF-κB), TNF-α, interferon gamma (IFN-γ)) and oxidative stress [36]. Moreover, Geng et al. [37] observed that mice undergoing a high-fat diet containing caprylic and capric acids had low levels of fasting glycemia and improvements in glucose tolerance and insulin sensitivity. Authors attributed these benefits to the anti-inflammatory effects of MCTs since they downregulated the expression of IL-6 and other biomarkers of inflammation [37]. The negative correlation of this compound with IBS-SSS score could also have a role in the improvement of symptoms in IBS-D patients. 

Few, if any, data are available on the metabolomic profile of IBS patients undergoing nutritional treatment, and most information on fecal VOCs derives from inflammatory bowel disease (IBD) studies. In this context, the presence of positive correlations between IBS-SSS and some VOCs related to inflammation, such as nonanal, benzaldehyde, ethanol, acetaldehyde, and the presence of a negative correlation between the IBS-SSS and the content in terpenes and MCFAs, for which a protective role has been hypothesized, seems to support our hypothesis on the potential role of VOCs in IBS-D symptomatology.

Moreover, in the present study, the hypothesis that the SCFAs and BCFAs in patients with IBS differed among the two groups was investigated. Previous research suggested that SCFAs showed an altered profile in IBS conditions [38,39,40]. In fact, our results highlighted unbalanced fecal organic acid levels in IBS groups without significant differences at baseline among TBD and LFD. Specifically, among the SCFAs analyzed, only acetic acid and propanoic acid decreased in IBS-D patients after treatments compared to the baseline (Figure 5A,B). It is well-recognized that a high content of SCFAs characterizes the IBS condition. In fact, in a paper by Tana et al. [41], it was demonstrated that 26 patients with IBS showed a statistically significant increase in both acetic and propanoic acid compared to healthy subjects, underling that this increase was related to an imbalance of the intestinal flora caused by a significant increase of two bacterial species, *Veillonella* and *Lactobacillus*. Furthermore, as demonstrated by our results, after 12 weeks of treatment with TBD and LFD, acetic acid and propionic acid levels reached a lower value that significantly differed from that in TBD T_0_ and LFD T_0_. Moreover, in agreement with the study mentioned above [41], no difference was observed in the concentration of other BCFAs (such as isobutyric acid and isovaleric acid) in IBS patients.

Although we inferred important conclusions on VOC distribution in our patient cohort, to the best of our knowledge, this study presents some weaknesses. Firstly, we are conscious of the introduced bias relying on the final small cohort size. Secondly, a more prolonged intervention phase has to be tested to check if prolonged administration may affect the metabolites’ presence and abundance. Long-term studies involving a large population should be addressed to solidify the present results. Lastly, performing an appropriate 16S meta-taxonomic analysis of bacterial populations would be helpful. Their genomic potential in terms of biochemical metabolic pathways in the GI tract would greatly aid in describing the observed change in the metabolome profiles.

## 5. Conclusions

The current literature broadly supports the hypothesis that disturbances in gut homeostasis exacerbate IBS symptoms through multiple mechanisms, in which the structure and function of the microbiome are essential contributors. Notwithstanding, the functional dynamics of gut microbiota and the specific effects of different nutritional approaches on this functional disorder are still to be investigated in many aspects. Here, we report the preliminary results from a pilot study aimed at comparing two diet regimens that have proven effective in reducing the symptoms of IBS-D patients on their metabolomic profile. Present findings demonstrate that these diets positively modified, although only with a few significant differences, the microbiome functions. Further research for a longer time is warranted to investigate the potentialities of TBD and LFD in managing IBS-D patients.

## Figures and Tables

**Table 1:** Baseline anthropometric characteristics of IBS-D patients categorized according to treatment.

Variable	IBS-D Patients TBD (*n* = 20)	IBS-D Patients LFD (*n* = 18)	*p*
Age, years	45.70 ± 10.20	42.90 ± 10.30	0.404
Males/Females	3/17	4/14	
Weight (kg)	69.80 ± 12.80	64.40 ± 13.30	0.293
Height (m)	1.62 ± 0.07	1.63 ± 0.10	0.823
BMI (kg/m^2^)	26.60 ± 4.70	24.03 ± 4.02	0.110
Abdominal circumference (cm)	93.50 ± 10.80	86.10 ± 10.40	0.060
Waist circumference (cm)	83.30 ± 13.30	77.02 ± 11.80	0.131

Data are expressed as mean (M) ± standard deviation (SD) (number where appropriate) and analyzed using the Mann–Whitney test. All differences were considered significant at *p* < 0.05. Abbreviations: BMI, body mass index; IBS-D, irritable bowel syndrome with diarrhea; LFD, low-fermentable oligosaccharides, disaccharides, monosaccharides, and polyols (FODMAPs) diet; TBD, tritordeum-based diet.

**Table 2:** Daily nutritional information of the IBS-D patients before (T_0_) and after (T_1_) 12 weeks of tritordeum-based diet (TBD) and low-FODMAP diet (LFD).

	TBD T_0_ (*n* = 20)	TBD T_1_ (*n* = 20)	*p*	LFD T_0_ (*n* = 18)	LFD_1_ (*n* = 18)	*p*
Energy consumption (kcal)	2206 ± 75	2235 ± 76	0.103	2142 ± 89	2136 ± 89	0.967
Energy intake (kcal)	1907 ± 137	1500 ± 27	0.021	2174 ± 176	1689 ± 93	0.014
Basal metabolism (kcal)	1454 ± 25	1471 ± 26	0.068	1500 ± 43	1505 ± 45	0.631
Proteins (g)	73 ± 4.5	75 ± 1.4	0.641	82.6 ± 6.1	83.3 ± 4.6	0.832
Proteins (%)	17 ± 0.4	20 ± 0.1	<0.0001	16 ± 0.4	19 ± 0.2	<0.0001
Lipids (g)	90 ± 8.9	50 ± 0.9	<0.0001	94.1 ± 10.0	55 ± 3.4	0.0007
Lipids (%)	42 ± 1.5	30 ± 0.1	<0.0001	36.3 ± 1.2	30.8 ± 0.2	<0.0001
Carbohydrates (g)	192 ± 13	198 ± 4.1	0.615	229 ± 12	228 ± 14	0.609
Carbohydrates (%)	41 ± 1.8	50 ± 0.2	<0.0001	47.7 ± 1.3	50.2 ± 0.3	0.02
Alcohol (%)	0.76 ± 0.3	0.49 ± 0.2	0.989	0.8 ± 0.3	0.29 ± 0.3	0.232
Dietary fiber (g)	14 ± 1.0	15 ± 0.4	0.383	16 ± 0.9	16 ± 0.9	0.330

Data are expressed as M ± SEM; the *p*-value for each diet was determined by Wilcoxon signed-rank test; differences were considered significant at *p* < 0.05. Comparisons between the two diets were performed by the Mann–Whitney test.

## Data Availability

The datasets used and/or analyzed during the current study are available from the corresponding author on reasonable request.

## Supplementary Materials

The following supporting information can be downloaded at: https://www.mdpi.com/article/10.3390/nu14214628/s1, Table S1: Concentration expressed as mean (sem) of volatile organic compounds (VOCs) detected in faecal samples from irritable bowel syndrome (IBS) with diarrhea (IBS-D) subjects at the baseline (T_0_) and after treatment (T_1_) undergoing Tritordeum-Based diet (TBD) and Low-FODMAP diet (LFD).
