# Supplementary material for: Evaluation of the Effects of the Tritordeum-Based Diet Compared to the Low-FODMAPs Diet on the Fecal Metabolome of IBS-D Patients: A Preliminary Investigation
Source: Nutrients. 2022 Nov 2;14(21):4628. doi: 10.3390/nu14214628 (PMC9657445; doi:10.3390/nu14214628)
Supplement: Supplementary file 1 [file nutrients-14-04628-s001.zip › nutrients-1978376-supplementary.pdf]

Table S1. Concentration expressed as mean (sem) of volatile organic compounds (VOCs) detected in faecal samples from irritable bowel syndrome (IBS) with diarrhea (IBS-D) subjects at the baseline (T_0) and after treatment (T_1) undergoing Tritordeum-Based diet (TBD) and Low-FODMAP diet (LFD).

	**TBD-T_0**	**TBD-T_1**	**LFD-T_0**	**LFD-T_1**
	μg/g *	μg/g	μg/g	μg/g
Ethanol	0.01(0.01)	0.14(0.073)	0.61(0.503)	0.14(0.077)
1-Butanol	0.03(0.022)	0.04(0.023)	0.04(0.029)	0.04(0.02)
1-Butanol, 2-methyl-,	0.11(0.046)	0.03(0.011)	0.1(0.039)	0.07(0.024)
1-Butanol, 3-methyl-	0.14(0.061)	0.05(0.021)	0.16(0.068)	0.08(0.024)
1-Pentanol	0.15(0.096)	0.04(0.015)	0.03(0.013)	0.1(0.031)
Phenethyl alcohol	1(0.142)	0.98(0.182)	1.39(0.276)	1.06(0.167)
1-Hexadecanol	2.48(0.568)	3.68(1.059)	1.56(0.355)	1.44(0.26)
Acetaldehyde	0.79(0.165)	0.91(0.234)	1.03(0.191)	0.92(0.199)
Propanal, 2-methyl- & Butanal	1.13(0.179)	1.03(0.157)	0.9(0.204)	1.07(0.215)
Butanal, 3-methyl-	3.26(0.637)	2.48(0.334)	2.76(0.567)	3.27(0.731)
Heptanal	0.03(0.017)	0.01(0.005)	0.11(0.072)	0.01(0.004)
2-Octenal, (E)-	0.35(0.343)	0.02(0.014)	0.08(0.042)	0.03(0.009)
Benzaldehyde	2.67(0.461)	1.91(0.192)	2.74(0.535)	2.24(0.337)
Benzeneacetaldehyde	0.54(0.151)	0.71(0.288)	0.39(0.112)	0.52(0.092)
Pentadecanal-	0.14(0.067)	0.09(0.03)	0.15(0.061)	0.16(0.102)
Nonanal	0.13(0.043)	0.1(0.034)	0.25(0.112)	0.03(0.016)
Acetic acid, methyl ester	n.d.	n.d.	0.07(0.058)	0.06(0.043)
Ethyl Acetate	0.01(0.008)	0.01(0.011)	0.11(0.07)	0.11(0.097)
Methyl propionate	n.d.	n.d.	0.07(0.049)	0.07(0.056)
Propanoic acid, ethyl ester	0.04(0.013)	0.05(0.02)	0.23(0.11)	0.18(0.136)
n-Propyl acetate	0.02(0.021)	0.02(0.023)	0.07(0.05)	0.08(0.071)
Butanoic acid, methyl ester	0.05(0.034)	0.12(0.094)	0.26(0.14)	0.46(0.382)
Methyl isovalerate	0(0.003)	0.01(0.008)	0.08(0.04)	0.03(0.019)
Butanoic acid, ethyl ester	0.41(0.193)	0.38(0.234)	0.77(0.225)	1.96(1.713)
Propanoic acid, propyl ester	0.11(0.047)	0.2(0.101)	0.24(0.083)	0.33(0.249)
Butanoic acid, 2-methyl-, ethyl ester	0.03(0.015)	0.05(0.029)	0.19(0.06)	0.05(0.02)
Acetic acid, butyl ester	0.07(0.05)	0.06(0.061)	0.02(0.017)	0.06(0.057)
Butanoic acid, 3-methyl-, ethyl ester	0.06(0.025)	0.04(0.024)	0.2(0.079)	0.05(0.024)
Methyl valerate	0.02(0.01)	0.03(0.026)	0.08(0.058)	0.16(0.134)
Butanoic acid, propyl ester	0.35(0.198)	0.43(0.31)	0.4(0.123)	1.14(0.943)
Pentanoic acid, ethyl ester	0.14(0.067)	0.13(0.073)	0.39(0.159)	0.73(0.589)
Butanoic acid, 2-methyl-, propyl ester	0.03(0.015)	0.11(0.072)	0.21(0.076)	0.03(0.017)
Propanoic acid, butyl ester	0.17(0.105)	0.11(0.114)	0.11(0.062)	0.17(0.148)
Butanoic acid, 2-methylpropyl ester	0.03(0.015)	0.04(0.031)	0.08(0.038)	0.02(0.013)
Butanoic acid, butyl ester	1.49(1.241)	0.85(0.844)	0.6(0.327)	0.92(0.773)
Butyl 2-methylbutanoate	0.02(0.019)	0(0.003)	0.06(0.036)	0.04(0.026)
Hexanoic acid, ethyl ester	0.04(0.034)	0.02(0.014)	0.04(0.023)	0.06(0.039)
Butanoic acid, 3-methylbutyl ester	0.05(0.039)	0.02(0.013)	0.06(0.049)	n.d.
Butanoic acid, pentyl ester	0.29(0.257)	0.23(0.228)	0.06(0.052)	0.01(0.01)
Pentanoic acid, butyl ester	0.34(0.204)	0.25(0.187)	0.27(0.096)	0.39(0.287)
Hexanoic acid, propyl ester	0.06(0.035)	0.01(0.005)	0.13(0.054)	1.12(1.102)
Cyclohexanecarboxylic acid, ethyl ester	0.33(0.231)	0.14(0.112)	0.33(0.307)	0.07(0.049)
Hexanoic acid, butyl ester	0.07(0.045)	0.15(0.122)	0.14(0.072)	0.7(0.666)
Butanoic acid, hexyl ester	0.01(0.009)	0.11(0.103)	0.06(0.035)	0.28(0.225)

Cyclohexanecarboxylic acid, propyl ester	0.36(0.301)	0.15(0.116)	0.24(0.228)	0.07(0.067)
Benzeneacetic acid, ethyl ester	0.09(0.027)	0.08(0.031)	0.18(0.063)	0.12(0.04)
Furan, 2,3-dihydro-	0.01(0.012)	0.05(0.037)	0.02(0.014)	0.02(0.009)
Furan, 3-(4-methyl-3-pentenyl)	0.06(0.02)	0.03(0.029)	0.04(0.019)	0.04(0.022)
Toluene	0.02(0.01)	0.02(0.009)	0.03(0.023)	0.02(0.007)
Dodecane	0.43(0.181)	0.11(0.042)	0.41(0.182)	0.28(0.102)
Tetradecane	0.31(0.087)	0.24(0.076)	0.27(0.052)	0.17(0.038)
2-Methyltetradecane	0.01(0.009)	0.01(0.009)	0(0.005)	0.03(0.015)
Pentadecane	0.19(0.047)	0.2(0.038)	0.2(0.049)	0.34(0.136)
Cyclopentadecane	0.86(0.345)	1.38(0.516)	1.9(1.426)	0.49(0.165)
7-Hexadecene, (Z)-	0.26(0.116)	0.08(0.037)	0.1(0.049)	0.06(0.036)
Pentadecane, 2,6,10,14-tetrame	0.01(0.009)	0.01(0.005)	0.08(0.065)	0.04(0.027)
Heptadecane	0.02(0.018)	0.09(0.046)	0.04(0.02)	0.08(0.057)
Nonadecane	0.13(0.042)	0.13(0.039)	0.12(0.047)	0.1(0.052)
Indole	23.3(2.979)	28.8(3.319)	17.3(2.603)	19.1(2.096)
Skatole	21.5(4.695)	16.5(3.321)	17.2(5.293)	19.5(5.361)
Ethyl acetone	0.2(0.038)	0.27(0.098)	0.2(0.058)	0.16(0.044)
Methyl Isobutyl Ketone	0.02(0.006)	0.03(0.009)	0.01(0.004)	0.02(0.007)
2-Pentanone, 3-methyl-	0.01(0.006)	0.03(0.02)	n.d.	0.01(0.009)
2,3-Pentanedione	0.05(0.033)	0.07(0.05)	0.04(0.032)	0.02(0.012)
2-Hexanone	0.04(0.029)	0.08(0.075)	0.05(0.034)	0.04(0.028)
3-Carene	0.21(0.094)	0.09(0.061)	0.16(0.054)	0.11(0.044)
Acetyl valeryl	0.02(0.013)	0.13(0.084)	0.04(0.035)	0.03(0.021)
5-Hepten-2-one, 6-methyl-	6.25(1.172)	5.5(2.642)	4.33(1.265)	5.72(1.442)
2-Undecanone	0.54(0.102)	0.58(0.129)	0.45(0.098)	0.34(0.085)
2-Dodecanone	0.11(0.04)	0.15(0.03)	0.12(0.033)	0.14(0.034)
2-Tridecanone	0.36(0.064)	0.48(0.108)	0.44(0.093)	0.46(0.067)
2-Tetradecanone	0.18(0.047)	0.22(0.071)	0.12(0.027)	0.1(0.022)
2-Pentadecanone	0.5(0.082)	0.45(0.065)	0.54(0.184)	0.34(0.045)
Dodecalactone	0.11(0.023)	0.08(0.016)	0.09(0.028)	0.08(0.026)
gamma-Dodecalactone	0.79(0.096)	0.65(0.128)	0.79(0.193)	0.63(0.137)
Pentanoic acid, 3-methylbutyl	0.02(0.009)	0.02(0.018)	0.05(0.037)	0.01(0.004)
Acetic acid	8.94(1.404)	6.6(1.491)	9.46(2.131)	6.88(2.718)
Propanoic acid	5.75(0.843)	5.27(1.076)	5.86(1.628)	4.16(1.167)
Isobutyric acid	2.69(0.406)	2.48(0.541)	2.41(0.598)	1.73(0.346)
Butanoic acid	20.9(5.209)	19.2(6.303)	17.9(3.433)	14.8(6.394)
Isovaleric acid	10.98(1.49)	11.7(2.881)	13.1(3.024)	8.93(1.912)
Pentanoic acid	10.5(1.649)	10.1(2.575)	10.1(2.076)	8.53(2.211)
Hexanoic acid	10.9(3.351)	11.3(2.91)	12.05(3.89)	13.2(7.011)
Nonanoic acid	2.24(0.835)	1.74(0.638)	1.54(0.38)	1.48(0.569)
n-Decanoic acid	0.13(0.091)	0.86(0.463)	0.36(0.268)	0.28(0.09)
Trimethylamine	n.d.	0.03(0.017)	0(0.002)	0.01(0.009)
(2E,4E)-3,7-Dimethylocta-2,4-diene	0.45(0.272)	0.17(0.065)	0.3(0.08)	0.35(0.117)
Geranyl isovalerate	0.11(0.061)	0.04(0.028)	0.12(0.085)	0.05(0.034)
Pyrazine, trimethyl-	0.03(0.011)	0.15(0.072)	0.04(0.013)	0.07(0.03)
Copaene	0.31(0.122)	0.03(0.019)	0.26(0.164)	0.25(0.099)
Pyrazine, tetramethyl-	0.01(0.014)	0.67(0.428)	n.d.	0.24(0.211)
Pyrazine,ethyl methyl	0.15(0.091)	0.86(0.523)	0.17(0.099)	0.36(0.252)
1,5,9-Undecatriene, 2,6,10-trimethyl	0.15(0.116)	0.16(0.068)	0.06(0.05)	0.21(0.163)
Estragole	1.67(1.435)	2.63(1.664)	1.5(1.444)	2.29(2.255)
Caparratriene	1.61(0.444)	2.47(1.232)	1.22(0.293)	1.09(0.383)

Anethole	0.52(0.353)	0.25(0.215)	0.4(0.312)	1.14(1.137)
5,9-Undecadien-2-one, 6,10-dimethyl-	0.6(0.117)	0.7(0.146)	0.64(0.171)	0.45(0.084)
trans-beta-Ionone	0.11(0.035)	0.24(0.074)	0.07(0.038)	0.18(0.044)
Ionone	0.05(0.036)	0.05(0.047)	n.d.	0.02(0.021)
2-Piperidinone	0.58(0.289)	0.77(0.348)	0.59(0.211)	0.51(0.352)
1H-Pyrrole-2,5-dione, 3-ethyl-	0.75(0.142)	1.05(0.285)	0.78(0.184)	0.69(0.129)
Pyrrolidine, 1-(1-pentenyl)-	0.03(0.031)	0.04(0.041)	0.03(0.029)	0(0.004)
2-indolone	0.16(0.026)	0.16(0.026)	0.18(0.042)	0.12(0.023)
Carvacrol	1.11(0.868)	0.33(0.082)	0.32(0.1)	0.28(0.093)
Phenol	2.32(0.594)	3.23(1.243)	3.12(0.918)	3.41(1.425)
p-Cresol	78.1 (6.712)	74.9(5.497)	66.1(10.07)	65.2(7.152)
Thymol	1.23(1.014)	0.12(0.098)	0.08(0.082)	0.11(0.058)
alpha-Pinene	0.16(0.067)	0.25(0.183)	0.25(0.127)	0.17(0.083)
(-)-beta-Pinene	0.22(0.082)	2.2(2.085)	0.37(0.239)	0.39(0.279)
alpha-Phellandrene	0.03(0.016)	0.02(0.011)	0.01(0.01)	0.05(0.034)
D-Limonene	2.63(0.756)	12.3(10.66)	4.45(2.131)	2.59(0.852)
gamma-Terpinene	0.6(0.265)	0.31(0.213)	1.04(0.568)	1(0.895)
o-Cymene	0.13(0.086)	2.06(1.994)	n.d.	0.08(0.039)
Caryophyllene	1.69(0.856)	0.47(0.166)	1.9(0.796)	1.69(1.113)
cis-?-Bergamotene	2.43(0.92)	1.15(0.444)	1.82(0.606)	1.79(1.151)
Humulene	0.13(0.082)	0.04(0.041)	0.11(0.058)	0.2(0.188)
Valencene	0.67(0.238)	0.21(0.134)	0.43(0.221)	1.1(0.738)
alpha-Muurolene	0.14(0.071)	0.02(0.018)	0.04(0.038)	0.77(0.742)
Zingiberene	0.76(0.631)	0.02(0.009)	0.14(0.136)	0.71(0.709)
beta-Bisabolene	0.55(0.225)	0.24(0.09)	0.43(0.194)	0.44(0.286)
Citral	0.38(0.112)	0.22(0.098)	0.15(0.055)	0.21(0.07)
Alpha-Farnesene	8.88(8.417)	1.66(1.032)	0.5(0.291)	0.15(0.077)
Beta-Sesquiphellandrene	0.28(0.25)	0.01(0.006)	0.05(0.048)	0.34(0.342)
alpha-Curcumene	0.61(0.452)	n.d.	0.08(0.079)	0.3(0.303)
2-Hexadecene, 3,7,11,15-tetramethyl-	0.13(0.043)	0.12(0.036)	0.11(0.055)	0.04(0.019)
Squalene	0.07(0.033)	0.05(0.022)	0.07(0.039)	0.03(0.009)

* VOC concentrations expressed as μg/g of internal standard. n.d. not detected.
